# How to schedule VEGF and PD-1 inhibitors in combination cancer therapy?

**DOI:** 10.1186/s12918-019-0706-y

**Published:** 2019-03-13

**Authors:** Xiulan Lai, Avner Friedman

**Affiliations:** 1 Institute for Mathematical Sciences, Renmin University of China, Beijing, People’s Republic of China; 2 Mathematical Bioscience Institute & Department of Mathematics, Ohio State University, Columbus, OH USA

**Keywords:** Anti-PD-1, Anti-VEGF, Combination therapy, Scheduling, PDE model

## Abstract

**Background:**

One of the questions in the design of cancer clinical trials with combination of two drugs is in which order to administer the drugs. This is an important question, especially in the case where one agent may interfere with the effectiveness of the other agent.

**Results:**

In the present paper we develop a mathematical model to address this scheduling question in a specific case where one of the drugs is anti-VEGF, which is known to affect the perfusion of other drugs. As a second drug we take anti-PD-1. Both drugs are known to increase the activation of anticancer T cells. Our simulations show that in the case where anti-VEGF reduces the perfusion, a non-overlapping schedule is significantly more effective than a simultaneous injection of the two drugs, and it is somewhat more beneficial to inject anti-PD-1 first.

**Conclusion:**

The method and results of the paper can be extended to other combinations, and they could play an important role in the design of clinical trials with combination therapy, where scheduling strategies may significantly affect the outcome.

## Background

Anti-vascular endothelial growth factor (anti-VEGF) is a drug commonly used as anticancer agent, although numerous studies show only modest results [1]. In combination with chemotherapy anti-VEGF improves anti-cancer therapy, although the outcome depends on the cancer type and on the scheduling of the treatment [2]. The scheduling issue arises from the fact that anti-VEGF decreases perfusion of chemotoxic agents in some cancers, including melanoma [3], breast cancer [4, 5] and ovarian cancer [6], while it increases perfusion of chemotoxic agents in other cancers, such as colon cancer [7, 8] and head and neck cancer [9]. More recently Asrid et al. [10] reported a rapid decrease in the delivery of chemotherapy to the tumor in patients of non-small cell lung cancer (NSCLC) after anti-VEGF therapy, highlighting the importance of drug scheduling in combination therapy when anti-VEGF is one of the drugs.

Less than 4% of positive phase II cancer clinical trials with combination chemotherapy demonstrate improvement of care in phase III [11]. Hence, the decision to go from phase II to phase III needs to identify more effectively which combinations will have a higher probability of success in phase III [12]. It was suggested in [13] that the design of clinical trials with combination therapy should be based, among other factors, on the scientific rationale underlying data and hypothesis for the combination.

In a previous work [14] we considered a combination therapy with a checkpoint inhibitor and cancer vaccine, and explored the synergy between the two drugs, taking into account potential negative side effects. In another paper [15] we considered the combination of BRAF/MEK inhibitor and checkpoint inhibitor, and showed that although the two drugs are positively correlated for most combinations of the doses, there is an exceptional range of doses where the two drugs are mutually antagonistic.

In the present paper we consider a combination therapy of anti-VEGF and a checkpoint inhibitor, and focus on the scheduling issue of these drugs. The rationale for using such a combination originates from the fact that VEGF impairs the function of anti-cancer T cells [16–20]; hence VEGF inhibition will enhance T cells function, and checkpoint blockade could therefore significantly advance antitumor therapy. The anticancer synergy between anti-VEGF and checkpoint inhibitors is currently being evaluated in clinical trials in renal cancer [21, 22].

In the case where anti-VEGF therapy decreases the perfusion of a second antitumor agent, the following question arises [23, 24]: Should the treatment with combination of anti-VEGF and a checkpoint inhibitor be given at the same time, or is it more beneficial to delay treatment of one of the two drugs, so that they are given non-overlappingly?

**Fig. 1:**
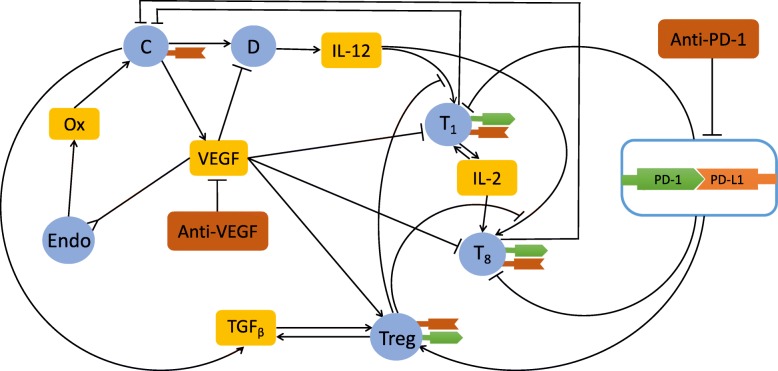
Interaction of immune cells with cancer cells. Sharp arrows indicate proliferation/activation, blocked arrows indicate killing/blocking, and the inverted arrow indicates recruitment/chemoattraction. C: cancer cells, D: dentritic cells, *T*_1_: CD 4^+^ Th1 cells, *T*_8_: CD 8^+^ T cells, Treg: T regulatory cells, Endo: endothelial cells, Ox: Oxygen from the blood. *T*_1_ and *T*_8_ cells and Tregs express PD-1 and PD-L1; tumor expresses PD-L1

To address this question we develop a mathematical model using a system of partial differential equations (PDEs). The variables of the model include CD 4^+^ (Th1) and CD 8^+^ T cells, regulatory T cells (Tregs), dendritic cells (DCs), endothelial cells, and cancer cells. The model also includes VEGF and TGF- *β* produced by cancer cells, and cytokines IL-12 and IL-2. The network of interactions among these species is shown in Fig. 1. This figure includes also oxygen concentration, and programmed cell death protein 1 (PD-1) and its ligand PD-L1. As indicated in Fig. 1. VEGF impairs the maturation of (antigen-presenting) dendritic cells [25, 26], and it suppresses the functions of activated T cells [16–20]; VEGF also enhances the expression of PD-1 on CD 8^+^ T cells [27], and induces Treg proliferation [28].

The mathematical model is based on the network shown in Fig. 1. The basic assumption in the model is that anti-VEGF decreases the perfusion of anti-PD-1 in the tumor microenvironment as in the case in melanoma, breast cancer, and ovarian cancer. The range of the injected amounts of drugs in the model was chosen so that the simulations will be in agreement with experimental results in mice models [27, 29].

We can use the model to assess the efficacy of the combination therapy when the treatment with anti-VEGF is delayed or advanced relative to anti-PD-1, for different schedules if treatment. In particular we show that the treatment is significantly more effective if instead of administering the two drugs at the same time, we administer them non-overlappingly.

We finally consider briefly the case where anti-VEGF increases the perfusion of anti-PD-1, and show that in this case there are more benefits when the two drugs are given simultaneously.

## Methods

### Mathematical model

The mathematical model is based on the network shown in Fig. 1. The list of variables is given in Table 1.

**Table 1:** List of variables (in units of g/ cm^3^)

Notation	Description
*D*	density of dendritic cells
*T*_1_	density of activated CD 4^+^ T cells
*T*_8_	density of activated CD 8^+^ T cells
*T*_*r*_	density of activated Treg cells
*E*	density of endothelial cells
*C*	density of cancer cells
*N*_*C*_	density of necrotic cell
*H*	HMGB-1 concentration
*I*_12_	IL-12 concentration
*I*_2_	IL-2 concentration
*T*_*β*_	TGF- *β* concentration
*W*	oxygen concentration
*G*	VEGF concentration
*P*_1_	concentration of PD-1 on CD 4^+^ T cells
*P*_8_	concentration of PD-1 on CD 8^+^ T cells
*L*	PD-L1 concentration
*Q*	PD-1-PD-L1 concentration
*A*	anti-PD-L1 concentration
*B*	anti-VEGF concentration

We assume that the total density of cells within the tumor remains constant throughout the tumor tissue, for all time:

1 $$  D+T_{1}+T_{8}+T_{r}+E+C=\text{constant},  $$

and that the density of debris of dead cells is also constant. We further assume that the densities of immature dendritic cells, and of naive CD 4^+^ and CD 8^+^ T cells, remain constant throughout the tumor tissue. As cancer cells proliferate, they “push away,” or displace, other cells. There is also migration of endothelial cells and immune cells into the tumor. Since the total density of cells was assumed to be constant at each point and time, by Eq.(1), these increases in the population of cells create a pressure (*p*) among the cells with an associated velocity field **u**. Under some additional assumptions on the material structure of the tumor, one can actually connect **u** to *p* (for example, by Darcy’s law in porous media), but we shall not need to do this in our model. The vector **u** is a function of space and time, taken in units of cm/day.

We also assume that all the cytokines and anti-tumor drugs are diffusing within the tumor tissue, and that also the cells are undergoing diffusion (i.e. dispersion), although with much smaller coefficients.

**Fig. 2:**
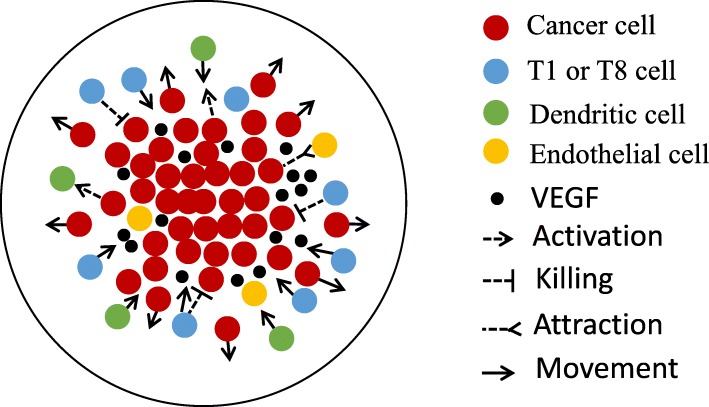
Distribution of cells in space

Although in our model we use densities of cells, it is interestingly to visualize how individual cells interact within the tumor. Figure 2 displays a distribution of cells in space, based on Fig. 1. We note, in particular, that cancer cells move toward the tumor boundary where the oxygen level can supports their abnormal proliferation; hence, by Eq. (1), the other types of cells are “pushed” toward the tumor core.

#### Equation for DCs (*D*)

By necrotic cancer cells (*N*_*C*_) we mean cancer cells undergoing the process of necrosis. Necrotic cancer cells release high mobility group box 1 protein (HMGB-1) [30]. We model the dynamics of *N*_*C*_ and HMGB-1 (*H*) by the following equations:

$$ {\begin{aligned} &\frac{\partial N_{C}}{\partial t}+\underbrace{\nabla \cdot (\mathbf{u} N_{C})}_{\text{velocity}}-\underbrace{\delta_{N_{C}}\nabla^{2} N_{C}}_{\text{difusion}}=\underbrace{\lambda_{N_{C}C}C}_{\text{derived\ from\ life\ cancer\ cells }}-\underbrace{d_{N_{C}}N_{C}}_{\text{removal}},\\ &\frac{\partial H}{\partial t}-\underbrace{\delta_{H}\nabla^{2} H}_{\text{difusion}}=\underbrace{\lambda_{HN_{C}}N_{C}}_{\text{released from nerotic cancer cells}}-\underbrace{d_{H}H,}_{\text{degradation}}\\ \end{aligned}} $$

where $\lambda _{N_{C}C}$ is the rate of cancer cells becoming necrotic and $\lambda _{HN_{C}}$ is the production rate of HMGB-1 by necrotic cells. We note that since molecules like HMGB-1, or other proteins, are several orders of magnitude smaller than cells, their diffusion coefficients are several orders of magnitude larger than the diffusion coefficients of cells, and they are only marginally influenced by the cells velocity *u*, so we do not include a velocity term in their equations. The degradation of HMGB-1 is fast (∼0.01/day) [31], and we assume that the process of necrosis is also fast. We may then approximate the two dynamical equations by the steady state equations $\lambda _{N_{C}C}C-d_{N_{C}}N_{C}=0$ and $\lambda _{HN_{C}}N_{C}-d_{H}H=0$, so that *H* is proportional to *C*.

Dendritic cells are activated by HMGB-1 [32, 33]. Hence, the activation rate of immature dendritic cells, with density *D*_0_, is proportional to $D_{0}\frac {H}{K_{H}+H}$, or to $D_{0}\frac {C}{K_{C}+C}$. Here, the Michaelis-Menten law was used to account for the limited rate of receptor recycling which takes place in the process of DCs activation. The dynamics of DCs is given by the following equation:

2 $$ {\begin{aligned}  \frac{\partial D}{\partial t}+\underbrace{\nabla \cdot (\mathbf{u} D)}_{\text{velocity}}-\underbrace{\delta_{D}\nabla^{2} D}_{\text{difusion}}=\underbrace{\lambda_{DC}D_{0}\frac{C}{K_{C}+C}}_{\text{activation by HMGB-1}}\cdot \underbrace{\frac{1}{1+G/K_{DG}}}_{\text{inhibition by VEGF}}-\underbrace{d_{D}D,}_{\text{death}} \end{aligned}}  $$

where *δ*_*D*_ is the diffusion coefficient, *d*_*D*_ is the death rate of DCs, and 1/(1+*G*/*K*_*DG*_) represents the impairment of maturation of dendritic cells by VEGF [25, 26].

#### Equations for CD 4^+^ T cells (*T*_1_) and CD 8^+^ T cells (*T*_8_)

Naive CD 4^+^ T cells differentiate into active Th1 cells (*T*_1_) under IL-12 (*I*_12_) environment [34, 35], and this process is inhibited by Tregs [36, 37] and by VEGF [16–20]. The proliferation of activated *T*_1_ cells is enhanced by IL-2. Both processes of activation and proliferation of *T*_1_ are assumed to be inhibited by PD-1-PD-L1 (*Q*) [38, 39]; we represent this inhibition by the factor $\frac {1}{1+Q/K_{TQ}}$. Hence *T*_1_ satisfies the following equation:

3 $$ {\begin{aligned}  \frac{\partial T_{1}}{\partial t} &+\nabla \cdot (\mathbf{u} T_{1})-\delta_{T}\nabla^{2} T_{1}\\&\,\,\,\,= \underbrace{\left(\lambda_{T_{1}I_{12}}T_{10}\cdot \frac{I_{12}}{K_{I_{12}}+I_{12}}\right.}_{\text{activation by IL-12}}\cdot \underbrace{\frac{1}{1+T_{r}/K_{TT_{r}}}}_{\text{inhibition by Tregs}}\cdot \underbrace{\frac{1}{1+G/K_{TG}}}_{\text{inhibition by VEGF}} \\ &+\underbrace{ \left. \lambda_{T_{1}I_{2}}T_{1}\frac{I_{2}}{K_{I_{2}}+I_{2}}\right)}_{\text{IL-2-induced proliferation}}\times \underbrace{\frac{1}{1+Q/K_{TQ}}}_{\text{inhibition by PD-1-PD-L1}}-\underbrace{d_{T_{1}}T_{1},}_{\text{death}} \end{aligned}}  $$

where *T*_10_ is the density of the naive CD 4^+^ T cells.

Similarly, inactive CD 8^+^ T cells are activated by IL-12 [34, 35], a process resisted by Tregs [36, 37] and VEGF [16–20], while IL-2 enhances the proliferation of activated CD 8^+^ T cells. Hence,

4 $$ {\begin{aligned}  \frac{\partial T_{8}}{\partial t}&+\nabla \cdot (\mathbf{u} T_{8})-\delta_{T}\nabla^{2} T_{8}\\&\,\,\,\,=\underbrace{\left(\lambda_{T_{8}I_{12}}T_{80}\cdot \frac{I_{12}}{K_{I_{12}}+I_{12}}\right.}_{\text{activation by IL-12}}\cdot \underbrace{\frac{1}{1+T_{r}/K_{TT_{r}}}}_{\text{inhibition by Tregs}}\cdot \underbrace{\frac{1}{1+G/K_{TG}}}_{\text{inhibition by VEGF}}\\ &+\underbrace{\left.\lambda_{T_{8}I_{2}}T_{8}\frac{I_{2}}{K_{I_{2}}+I_{2}}\right)}_{\text{IL-2-induced proliferation}} \times \underbrace{\frac{1}{1+Q/K_{TQ}}}_{\text{inhibition by PD-1-PD-L1}}-\underbrace{d_{T_{8}}T_{8},}_{\text{death}} \end{aligned}}  $$

where *T*_80_ is the density of the inactive CD 8^+^ T cells.

#### Equation for Tregs (*T*_*r*_)

Naive CD 4^+^ T cells differentiate into *T*_*r*_ cells under the influence of TGF- β: (*T*_*β*_) [37, 40] and VEGF [28]. The equation for *T*_*r*_ takes the following form:

5 $$ \begin{aligned}  \frac{\partial T_{r}}{\partial t}+\nabla \cdot (\mathbf{u} T_{r})-\delta_{T}\nabla^{2} T_{r}=&T_{10}\underbrace{\left(\lambda_{T_{r}T_{\beta}}\frac{T_{\beta}}{K_{T_{\beta}}+T_{\beta}}\right.}_{\text{TGF}-\beta-\text{induced proliferation}}\\&+\underbrace{\lambda_{T_{r}G}\left.\frac{G}{K_{G}+G}\right)}_{\text{promotion by VEGF}}-\underbrace{d_{T_{r}}T_{r}.}_{\text{death}} \end{aligned}  $$

#### Equation for endothelial cells (*E*)

Endothelial cells are chemoattracted by VEGF, and their proliferation is increased by VEGF [41, 42]. The equation for the density of endothelial cells is given by

6 $$ \begin{aligned}  \frac{\partial E}{\partial t}+\nabla \cdot (\mathbf{u} E)-\delta_{E}\nabla^{2} E=&\underbrace{\lambda_{E}(G)E\left(1-\frac{E}{E_{M}}\right)}_{\text{proliferation}}\\&-\underbrace{\nabla \cdot (\chi_{G} E\nabla G)}_{\text{recruited by VEGF}}-\underbrace{d_{E}E,}_{\text{death}} \end{aligned}  $$

where *E*_*M*_ is the carrying capacity of endothelial cells, *λ*_*E*_(*G*)=*λ*_*EG*_(*G*−*G*_0_)^+^, and *G*_0_ is a threshold below which endothelial cells do not proliferate [43]. Here we used the notion: *X*^+^=*X* if *X*≥0 and *X*^+^=0 if *X*<0.

#### Equation for cancer cells (*C*)

We assume a logistic growth for cancer cells, with carrying capacity (*C*_*M*_), in order to account for competition for space among these cells. The proliferation rate depends on the density of oxygen (*W*) [42]. The equation for *C* takes the following form:

7 $$ \begin{aligned}  \frac{\partial C}{\partial t}+\nabla \cdot (\mathbf{u} C)-\delta_{C}\nabla^{2} C=&\underbrace{\lambda_{C}(W)C\left(1-\frac{C}{C_{M}}\right)}_{\text{proliferation}}\\*&-\underbrace{\eta_{1}T_{1}C-\eta_{8} T_{8}C}_{\text{killing by T cells}}-\underbrace{d_{C}C,}_{\text{death}} \end{aligned}  $$

where *η*_1_ and *η*_8_ are the killing rates of cancer cells by *T*_1_ and *T*_8_ cells, respectively. *d*_*C*_ is the natural death rate of cancer cells, and

$$\lambda_{C}(W)=\left\{ \begin{array}{ll} \lambda_{CW}\frac{W}{W_{0}} & \,\,\text{if}\:\: W\le W_{0}\\ \lambda_{CW} & \,\,\text{if}\:\: W> W_{0}, \end{array} \right. $$

where *W*_0_ is the normal level of oxygen concentration in the blood.

#### Equation for IL-12 (*I*_12_)

The proinflammatory cytokine IL-12 is secreted by activated DCs [34, 35]; hence it satisfies the equation:

8 $$\begin{array}{@{}rcl@{}}  \frac{\partial I_{12}}{\partial t}-\delta_{I_{12}}\nabla^{2} I_{12}&=&\underbrace{\lambda_{I_{12}D}D}_{\text{production by DCs}}-\underbrace{d_{I_{12}}I_{12}.}_{\text{degradation}} \end{array} $$

#### Equation for IL-2 (*I*_2_)

IL-2 is produced by activated CD 4^+^ T cells (*T*_1_) [35]. Hence,

9 $$\begin{array}{@{}rcl@{}}  \frac{\partial I_{2}}{\partial t}-\delta_{I_{2}}\nabla^{2} I_{2}&=&\underbrace{\lambda_{I_{2}T_{1}}T_{1}}_{\text{production by \(T_{1}\)}}-\underbrace{d_{I_{2}}I_{2}.}_{\text{degradation}} \end{array} $$

#### Equation for TGF-β: (*T*_*β*_)

TGF-β is produced by tumor cells [36] and Tregs [37], so that

10 $$ \begin{aligned}  \frac{\partial T_{\beta}}{\partial t}-\delta_{T_{\beta}}\nabla^{2} T_{\beta}=&\underbrace{\lambda_{T_{\beta}C}C}_{\text{production by cancer cells}}\\&+\underbrace{\lambda_{T_{\beta}T_{r}}T_{r}}_{\text{production by Tregs}}-\underbrace{d_{T_{\beta}}T_{\beta}.}_{\text{degradation}} \end{aligned}  $$

#### Equation for oxygen (*W*)

Oxygen is infused through the blood [41, 42]. We identify the blood concentration with the density of endothelial cells and, accordingly, write the equation for *W* in the following form:

11 $$ \begin{aligned}  \frac{\partial W}{\partial t}-\delta_{W}\nabla^{2} W=&\underbrace{\lambda_{WE}E}_{\text{source from blood}}-\underbrace{d_{W}W,}_{\text{consumption by cells}} \end{aligned}  $$

where *d*_*W*_*W* represents the take-up rate of oxygen by all the cells.

#### Equation for VEGF (*G*)

VEGF is produced by cancer cells [41, 42] and is depleted by anti-VEGF. Hence *G* satisfies the following equation:

12 $$ \begin{aligned}  \frac{\partial G}{\partial t}-\delta_{G}\nabla^{2} G=&\underbrace{\lambda_{G}(W)C}_{\text{production by cancer cells}}- \underbrace{\mu_{GB}GB}_{\text{inhibition by anti-VEGF}}\\ &-\underbrace{d_{G}G}_{\text{degradation}}, \end{aligned}  $$

where *B* is the effective anti-VEGF concentration in the tumor, and

$$\lambda_{G}(W)=\lambda_{GW}\times \left\{ \begin{array}{ll} \frac{W}{W^{*}} & \,\,\text{if}\:\: 0\le W\le W^{*}\\ 1-0.7\frac{W-W^{*}}{W_{0}-W^{*}} & \,\,\text{if}\:\:W^{*}< W\le W_{0}\\ 0.3 & \,\,\text{if}\:\: W> W_{0}.\\ \end{array}\right. $$

Here we assumed that the secretion rate of VEGF by cancer cells increases with the oxygen level, but falls off when oxygen level exceeds a certain level, *W*^∗^ [44].

#### Equations for PD-1 (*P*), PD-L1 (*L*) and PD-1-PD-L1 (*Q*)

PD-1 is expressed on the surface of activated CD 4^+^ T cells, activated CD 8^+^ T cells, and Tregs [38, 45]. We assume that the number of PD-1 receptors per cell is the same for *T*_1_ and *T*_8_ cells, but is smaller, by a factor *ε*_*T*_, for *T*_*r*_ cells. VEGF increases the PD-1 on *T*_8_ cells by a factor *ε*_*G*_*G* [27]. If we denote by *ρ*_*P*_ the ratio between the mass of one PD-1 protein to the mass of one T cell, then the total concentration of PD-1 on *T*_1_ and *T*_*r*_ cells is given by

13 $$  P_{1}=\rho_{P}\left(T_{1}+\varepsilon_{T} T_{r}\right),  $$

and the concentration of PD-1 on *T*_8_ cells is given by

14 $$  P_{8}=\rho_{P}T_{8}(1+\varepsilon_{G}G).  $$

PD-L1 is expressed on activated CD 4^+^ T cells, activated CD 8^+^ T cells [38], Tregs [46], and cancer cells [38, 39]. We assume that the number of PD-L1 per cell is the same for *T*_1_ and *T*_8_ cells, and denote the ratio between the mass of one PD-L1 protein to the mass of one cell by *ρ*_*L*_. Then

$$L=\rho_{L}(T_{1}+T_{8}+\varepsilon_{T}T_{r}+\varepsilon_{C} C), $$

for some parameters *ε*_*T*_,*ε*_*C*_, where *ε*_*C*_ depends on the specific type of tumor.

To a change in *T*=*T*_1_+*ε*_*T*_*T*_*r*_, given by $\frac {\partial T}{\partial t}$, there corresponds a change of *P*_1_, given by $\rho _{P}\frac {\partial T}{\partial t}$. For the same reason, ∇·(**u***P*_1_)=*ρ*_*P*_∇·(**u***T*) and ∇^2^*P*_1_=*ρ*_*P*_∇^2^*T* when no anti-PD-1 drug is injected. Hence, *P*_1_ satisfies the following equation:

$$ \begin{aligned} \frac{\partial P_{1}}{\partial t}+\nabla \cdot (\mathbf{u} P_{1})-\delta_{T} \nabla^{2} P_{1}=&\rho_{P}\left[\frac{\partial(T_{1}+\varepsilon_{T}T_{r})}{\partial t} \right.\\ &+\nabla \cdot (\mathbf{u} (T_{1}+\varepsilon_{T}T_{r}))\\ &\left.-\delta_{T}\nabla^{2} (T_{1}+\varepsilon_{T}T_{r}){\vphantom{\frac{\partial(T_{1}+\varepsilon_{T}T_{r})}{\partial t}}}\right]. \end{aligned} $$

Recalling Eqs. (3) and (5) for *T*_1_ and *T*_*r*_, we get,

$$ \begin{aligned} \frac{\partial P_{1}}{\partial t}+&\nabla \cdot (\mathbf{u} P_{1})-\delta_{T} \nabla^{2} P_{1}\\&=\rho_{P} \left[\text{RHS of Eq.\:(3)}+\varepsilon_{T},\times \text{RHS of Eq.\:(5)} \right] \end{aligned} $$

where RHS=right-hand side. Similarly,

$$ {\begin{aligned} \frac{\partial P_{8}}{\partial t}+&\nabla \cdot (\mathbf{u} P_{8})-\delta_{T} \nabla^{2} P_{8}\\ &=\rho_{P} \left[(1+\varepsilon_{G}G)\times \text{RHS of Eq.\:(4)} + \varepsilon_{G} T_{8} \times\text{RHS of Eq.\:(12)}\right.\\ & \left.+ \varepsilon_{G}T_{8}\nabla\cdot\mathbf{u}G+\varepsilon_{G}(\delta_{G}-\delta_{T})T_{8}\nabla^{2}G-2\varepsilon_{G}\delta_{T}\nabla T_{8}\cdot \nabla G \right]. \end{aligned}} $$

When only anti-PD-1 drug (*A*) is injected, PD-1 is depleted at a rate *μ*_*PA*_*P*_1_*A*, but when also anti-VEGF (*B*) is injected the depletion of PD-1 is reduced, due to restricted perfusion, by a factor $\frac {1}{1+B/K_{PB}}$; since all other species of the model evolve within the tumor, we ignored the effect of restricted perfusion in setting up their dynamics. We conclude that *P*_1_ satisfies the following equation:

15 $$ \begin{aligned}  \frac{\partial P_{1}}{\partial t}+&\nabla \cdot (\mathbf{u} P_{1})-\delta_{T} \nabla^{2} P_{1}\\ &=\rho_{P}\left[\text{RHS of Eq.\:(3)} +\varepsilon_{T}\times \text{RHS of Eq.\:(5)}\right]\\ &-\underbrace{\mu_{PA} P_{1}A}_{\text{ depletion\ by\ anti-PD-1 }}\times \underbrace{\frac{1}{1+B/K_{PB}}.}_{\text{ blockade\ of\ perfusion\ by\ anti-VEGF }} \end{aligned}  $$

Similarly,

16 $$ {\begin{aligned}  \frac{\partial P_{8}}{\partial t}+&\nabla \cdot (\mathbf{u} P_{8})-\delta_{T} \nabla^{2} P_{8}\\&=\rho_{P}\left[(1+\varepsilon_{G}G)\times \text{RHS of Eq.\:(4)}\right.\\ & +\varepsilon_{G}T_{8} \times\text{RHS of Eq.\:(12)}\\ &\left. + \varepsilon_{G} T_{8}\nabla\cdot\mathbf{u}G+\varepsilon_{G}(\delta_{G}-\delta_{T})T_{8}\nabla^{2}G -2\varepsilon_{G}\delta_{T}\nabla T_{8}\cdot \nabla G \right] \\ & -\underbrace{\mu_{PA} P_{8}A}_{\text{ depletion\ by\ anti-PD-1 }}\times \underbrace{\frac{1}{1+B/K_{PB}}.}_{\text{ blockade\ of\ perfusion\ by\ anti-VEGF }} \end{aligned}}  $$

PD-L1 combines with PD-1 on the plasma membrane of T cells, to form a complex PD-1-PD-L1 (*Q*) [38, 39]. Denoting the association and disassociation rates of *Q* by *α*_*PL*_ and *d*_*Q*_, respectively, we can write

$$ P+L \overset{\alpha_{PL}}{\underset{d_{Q}}{\rightleftharpoons}} Q, $$

where *P*=*P*_1_+*P*_8_. The half-life of *Q* is less then 1 s (1.16×10^−5^day) [47], so that *d*_*Q*_ is very large. Hence we may approximate the dynamical equation for *Q* by the steady state equation, *α*_*PL*_*PL*=*d*_*Q*_*Q*, so that

17 $$  Q=\sigma PL,  $$

where *σ*=*α*_*PL*_/*d*_*Q*_.

#### Equation for anti-PD-1 (*A*)

We assume that the drug enters the tumor from the boundary and quickly diffuses, so that its concentration is a constant *γ*_*A*_ throughout the tumor during the dosing period. We denote by *μ*_*AP*_ the depletion rate of *A* caused by blocking PD-1. Hence,

18 $$ {\begin{aligned}  \frac{\partial A}{\partial t}-\delta_{A} \nabla^{2} A&=&\gamma_{A}I_{A}(t) -\underbrace{\mu_{AP} PA}_{\text{depletion through blocking PD-1}}-\underbrace{d_{A}A,}_{\text{degradation}} \end{aligned}}  $$

where *I*_*A*_(*t*)=1 during dosing, and *I*_*A*_(*t*)=0 otherwise.

#### Equation for anti-VEGF (*B*)

We denote by *γ*_*B*_ the concentration of the injected drug *B* during the dosing period, and by *μ*_*BG*_ the depletion rate of *B* blocking VEGF. The equation for *B* is then given by

19 $$ \begin{aligned} \frac{\partial B}{\partial t}-\delta_{B} \nabla^{2} B=&\gamma_{B}I(t) -\underbrace{\mu_{BG}GB}_{\text{depletion by VEGF}}-\underbrace{d_{B}B,}_{\text{degradation}} \end{aligned}  $$

where *I*_*B*_(*t*)=1 during dosing, and *I*_*B*_(*t*)=0 otherwise.

#### Equation for cells velocity (**u**)

We assume that the density of each cell type in the growing tumor tends to a steady state, and take the density of the extracellular matrix (ECM) in steady state to be 0.6g/cm^3^.

We take the steady state density of endothelial cells to be *E*=2.5×10^−3^ g/ cm^3^ [43]. The steady state densities of the immune cells *D*, *T*_1_,*T*_8_,*T*_*r*_ and *C* (in units of g/cm^3^) are taken to be (see Appendix: Parameter estimation)

20 $$  {\begin{aligned} D=4\times 10^{-4}, \ \ T_{1}=2\times 10^{-3},\ \ T_{8}=1\times 10^{-3}, \ \ T_{r}= 5\times 10^{-4}, \ \ C=0.4. \end{aligned}}  $$

With these choices, the constant in Eq. (1) equal to 0.4064. We further assume that all cells have approximately the same diffusion coefficient. Adding Eqs. (2)-(7), we get

21 $$  0.4064\times \nabla\cdot \mathbf{u}=\sum_{j=2}^{7} \left[\text{RHS of Eq.\:(j)} \right].  $$

To simplify the computations, we assume that the tumor is spherical and denote its radius by *r*=*R*(*t*). We also assume that all the densities and concentrations are radially symmetric, that is, they are functions of (*r*,*t*), where 0≤*r*≤*R*(*t*). In particular, **u**=*u*(*r*,*t*)**e**_*r*_, where **e**_*r*_ is the unit radial vector.

#### Equation for free boundary (*R*)

We assume that the free boundary *r*=*R*(*t*) moves with the velocity of the cells, so that

22 $$  \frac{dR(t)}{dt}=u(R(t),t).  $$

#### Boundary conditions

We assume that the naive CD 4^+^ T cells and CD 8^+^ T cells which migrated from the lymph nodes into the tumor microenvironment have constant densities $\hat T_{1}$ and $\hat T_{8}$ at the tumor’s boundary, and, that upon crossing the tumor boundary, *T*_1_ and *T*_8_ are activated by IL-12 and *T*_*r*_ is activated by *T*_*β*_. We then have the following flux conditions at the tumor’s boundary:

23 $$ {\begin{aligned}  &\frac{\partial T_{1}}{\partial r}+\sigma_{T}(I_{12})(T_{1}-\hat T_{1})=0,\:\: \frac{\partial T_{8}}{\partial r}+\sigma_{T}(I_{12})(T_{8}-\hat T_{8})=0,\\ &\frac{\partial T_{r}}{\partial r}+\sigma_{T_{r}}(T_{\beta})(T_{r}-\hat T_{1})=0\quad \text{at}\:\: r=R(t), \end{aligned}}  $$

where we take $\sigma _{T}(I_{12})=\sigma _{0} \frac {I_{12}}{K_{I_{12}}+I_{12}}$ and $\sigma _{T_{r}}(T_{\beta })=\sigma _{0} \frac {T_{\beta }}{K_{T_{\beta }}+T_{\beta }}$. We assume that the endothelial cells that are attracted by the VEGF into the tumor microenvironment have constant density $\hat E$ at the tumor’s boundary, and take

24 $$ \begin{aligned}  &\frac{\partial E}{\partial r}+\sigma_{E}\frac{G}{K_{G}+G}(E-\hat E)=0 \quad \text{at}\:\: r=R(t). \end{aligned}  $$

The boundary condition for the oxygen is given by

25 $$ \begin{aligned}  &\frac{\partial W}{\partial r}+\sigma_{W}(W-W_{0})=0 \quad \text{at}\:\: r=R(t), \end{aligned}  $$

where *W*_0_ is the normal level of oxygen concentration in the blood. We impose no-flux boundary condition for all the remaining variables:

26 $$  \begin{aligned} \text{No-flux for} &\:D,C,I_{12},I_{2},T_{\beta},G,P_{1},P_{8}, A,\:\text{and} \:B\: \text{at}\: r=R(t). \end{aligned}  $$

It is tacitly assumed here that the receptors PD-1 become active only after the T cells are already inside the tumor.

#### Initial conditions

We take the following initial values (in units of g/cm^3^):

27 $$ {\begin{aligned}  &D=2\times 10^{-3}, \: T_{1}\!=4\times 10^{-3},\:T_{8}\!=2\times 10^{-3}, \: T_{r}\!= 3\times 10^{-4}, \:E=2.45\times 10^{-3},\\ & C= 0.39565, \: I_{12}=2.88\times 10^{-9},\: I_{2}=4.74\times 10^{-11},\: T_{\beta}=2.57\times 10^{-13},\\ & W=1.52\times 10^{-4},\: G=6.3\times 10^{-8},\:P_{1}=1.06\times 10^{-9},\:P_{8}=5.43\times 10^{-11}, \\ & R(0)=0.01\:\text{cm}. \end{aligned}}  $$

Note that the total density of cells at time *t*=0 satisfies Eq. (1) with the already chosen constant 0.4064. We also mention that the choice of initial conditions does not affect the simulation results after a few days.

## Results

The simulations of the model were performed by Matlab, based on the moving mesh method for solving partial differential equations with free boundary [48] (see the section on computational method).

### Simulation results for mouse model

**Fig. 3:**
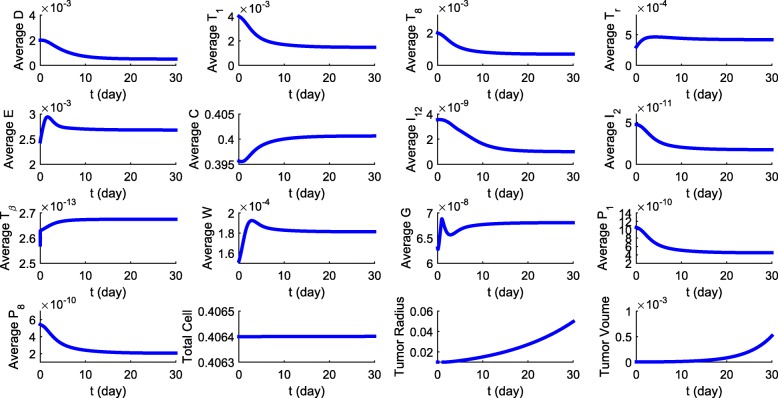
Average densities/concentrations, in g/cm^3^, of all the variables in the model with control case (no drugs). All parameter values are the same as in Tables 3 and 4, for a mouse model

Figure 3 shows the profiles of the average densities/concentrations of all the variables of the model in the control case (no drugs) and the tumor volume in the first 30 days. We note that each species *X* reaches a nearly steady state that is approximately the half-saturation value, *K*_*X*_, as assumed in the estimation of some of the model parameters (in Appendix). This shows the consistency in the parameters estimation.

From Fig. 3 we see that in the first few days *E*, and corresponding *W*, are increasing before they begin to gradually decrease and reach a steady state. The production rate of *G* is given by *λ*(*W*)*C* where *λ*(*W*) is bimodal and the oscillation in the profile of *G* is affected by the bimodal profile of *W* and the initial growth in the profile of *C*. The initial increase in *G* results in initial decrease in *D*, and hence also a decrease in IL-12 and the T cells. The profile of *C* is affected by the growth in *W* and by the decrease in *T*_1_ and *T*_8_: *C* begins to grow after a short time, but the growth rate decreases to near 0 as time increases.

**Fig. 4:**
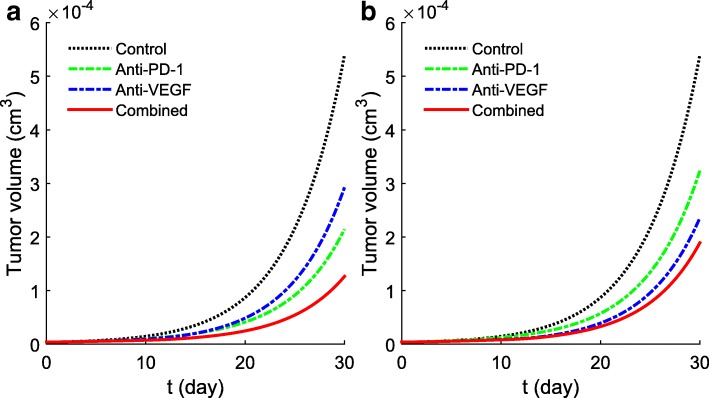
Growth of tumor volume under treatment with *γ*_*B*_ or *γ*_*A*_, or combination (*γ*_*B*_,*γ*_*A*_). The anti-VEGF or/and the anti-PD-1 treatment started at day 0 and continued for 10 days. **a** *γ*_*B*_=3×10^−8^g/cm^3^·day,*γ*_*A*_=3×10^−8^g/cm^3^·day; **b** *γ*_*B*_=3.5×10^−8^g/cm^3^·day,*γ*_*A*_=0.5×10^−8^g/cm^3^·day. All other parameters are same as in Fig. 3

**Fig. 5:**
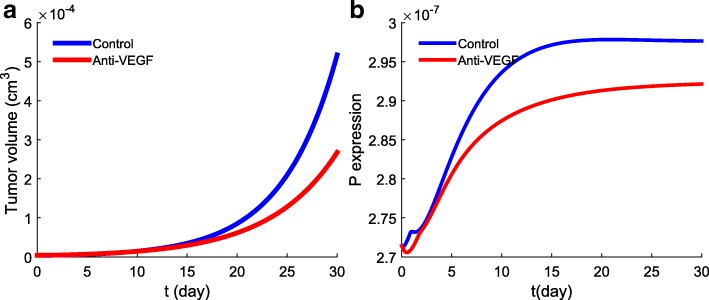
Anti-VEGF decreases PD-1 expression on CD 8^+^ T cells. The treatment with anti-PD-1 drug started at day 0 and continued for 30 days with *γ*_*B*_=2×10^−8^g/cm^3^·day (**a**) Growth of tumor volume. (**b**) Expression level of PD-1 on CD 8^+^ T cells

Before applying our model to clinical situations, we need to determine the order of magnitude of *γ*_*B*_ and *γ*_*A*_. The parameter *γ*_*B*_ is proportional to the amount of anti-VEGF administered to a patient, but it is difficult to determine this proportionality coefficient; the same is true for *γ*_*A*_. We therefore conducted simulations with different choices of *γ*_*B*_,*γ*_*A*_ in order to find values for which the simulations agree qualitatively with mice experiments. One set of simulations is displayed in Fig. 4. Figure 4a shows that with *γ*_*B*_=3×10^−8^g/cm^3^·day and *γ*_*A*_=3×10^−8^g/cm^3^·day the anti-PD-1 as a single agent reduces the volume of tumor more than anti-VEGF as a single agent, in agreement with Fig. 1 in [29]. Figure 4b shows that with *γ*_*B*_=3.5×10^−8^g/cm^3^·day and *γ*_*A*_=0.5×10^−8^g/cm^3^·day the anti-VEGF as a single agent reduces the tumor volume more than the anti-PD-1 as single agent, in agreement with Fig. 4j in [27]. In Fig. 5 we see that (with *γ*_*A*_=0) anti-VEGF with *γ*_*B*_=2×10^−8^g/cm^3^·day reduces significantly the PD-1 expression on CD 8^+^ T cells, in agreement with experimental results in [27].

### Clinical trials *in silico*

In clinical trials the treatment and follow-up periods are much longer than in mouse models, and the tumor growth is significantly slower. Accordingly, we shall modify some of the parameters in order to slow the growth of the tumor; these parameters are *λ*_*DC*_,*λ*_*E*_,*λ*_*CW*_. We shall also decrease the range of the drug, from order of magnitude 10^−8^ to 10^−9^−10^−10^.

We consider clinical treatment in cycles of 9 weeks, whereby each drug is given continuously for 3 weeks during a cycle with drug holiday for 6 weeks. We introduce three different schedules. In schedule S1 anti-PD-1 and anti-VEGF are both administered continuously in the first 3 weeks of the 9-week cycle. In schedule S2 anti-PD-1 is given continuously in the first 3 weeks, followed by anti-VEGF in the next 3 weeks, with no drugs for the remaining 3 weeks of the cycle. Schedule S3 is similar to schedule S2 with anti-VEGF in the first 3 weeks and anti-PD-1 in the next 3 weeks. We refer to schedules S2 and S3 as non-overlapping schedules.

**Fig. 6:**
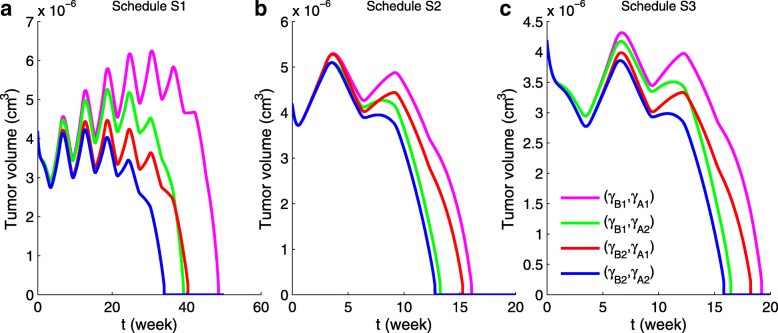
Tumor volume under schedules S1, S2 and S3 for 4 pairs (*γ*_*B*_,*γ*_*A*_). Here *γ*_*B*1_=9.5×10^−9^g/cm^3^·day,*γ*_*A*1_=1.2×10^−10^g/cm^3^·day,*γ*_*B*2_=10×10^−9^g/cm^3^·day,*γ*_*A*2_=1.5×10^−10^g/cm^3^·day.**a** Tumor volume under schedule S1; **b** Tumor volume under schedule S2; **c** Tumor volume under schedule S3

Figure 6 shows the profile of the tumor volume under schedules S1, S2 and S3 for four different dose pairs (*γ*_*B*_,*γ*_*A*_). Table 2 summarizes the time, in weeks, at which the tumor volume decreased by 95% from its initial size. We see that the non-overlapping schedules S2 and S3 reduce the tumor volume significantly faster than schedule S1, and schedule S2 is somewhat more effective than schedule S3.

**Table 2:** The time, in weeks, at which the tumor volume decreased to 5% of its initial size

Schedule	(*γ*_*B*1_,*γ*_*A*1_)	(*γ*_*B*1_,*γ*_*A*2_)	(*γ*_*B*2_,*γ*_*A*1_)	(*γ*_*B*2_,*γ*_*A*2_)
S1	48.59	39.17	40.4	33.99
S2	16.05	13.26	15.25	12.78
S3	19.22	16.46	18.24	15.83

**Fig. 7:**
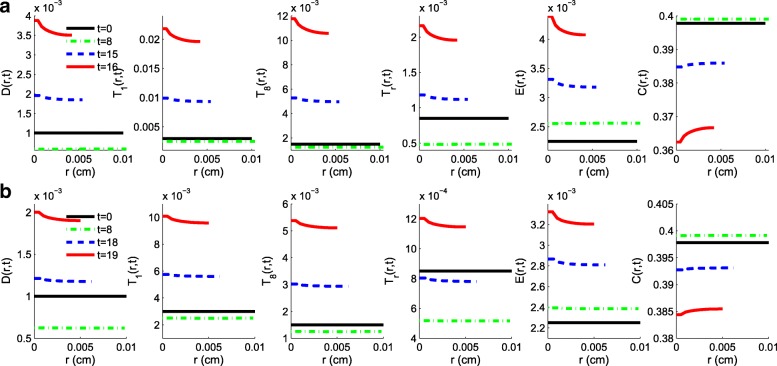
Spatial profiles of the densities of dendritic cells, T cells, endothelial cells and cancer cells with *γ*_*A*_=1.2×10^−10^g/cm^3^·day,*γ*_*B*_=9.5×10^−9^g/cm^3^·day. **a** Profiles under schedule S2 at day *t*=0,8,15 and 16 weeks. **b** Profiles under schedule S3 at days *t*=0,8,18 and 19 weeks

Figure 7 displays the density profiles of the immune cells, endothelial cells and cancer cells at different times along the radius of the tumor, with *γ*_*A*_=1.2×10^−10^g/cm^3^·day,*γ*_*B*_=9.5×10^−9^g/cm^3^·day. Figure 7a shows the profiles under schedule S2 at times *t*=0,8,15 and 16 weeks, and Fig. 7b shows the profiles under schedule S3 at times *t*=0,8,18 and 19 weeks. We see that the initially assumed flat density profiles are evolving to develop a distinct monotonic form. The density of cancer cells (*C*) is increasing from the tumor core to the tumor boundary where the level of oxygen (*W*) is more favorable to their abnormal proliferation. Since the total density of all the cells is constant (by Eq. (1)), the immune cells and the endothelial cells are “pushed” back toward the core of the tumor, so that their profile is monotone decreasing from the tumor core toward the tumor boundary.

**Fig. 8:**
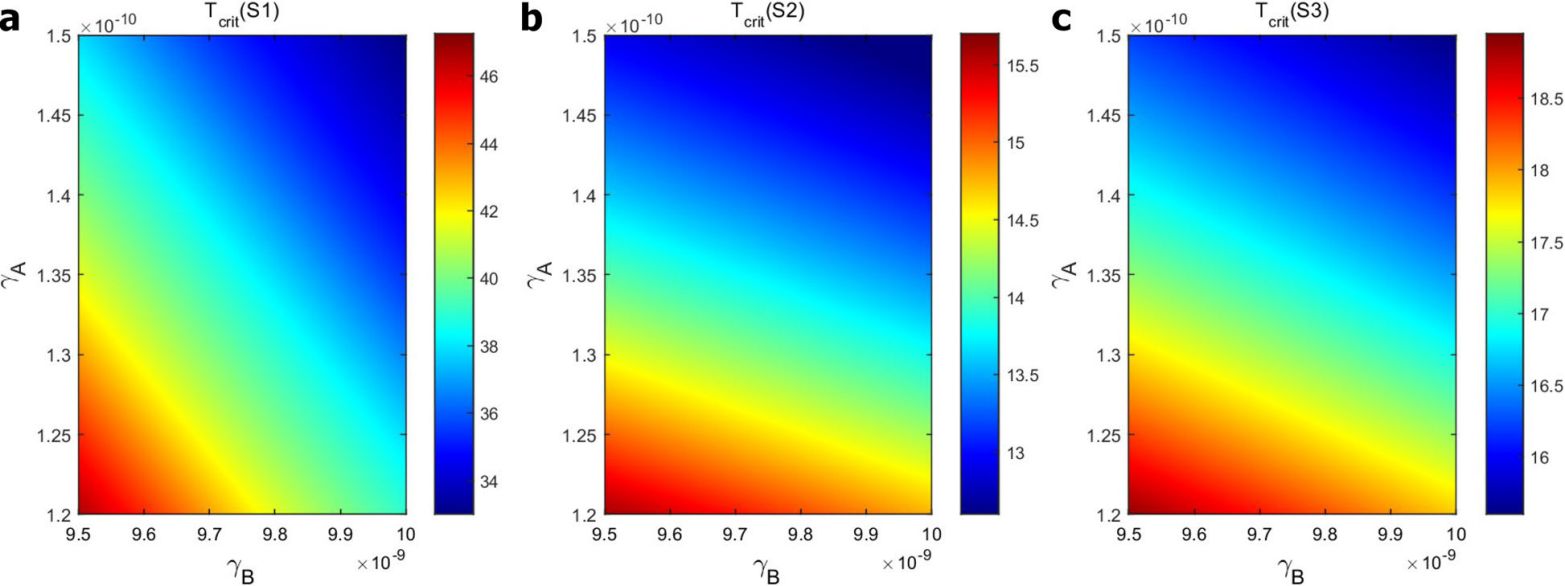
Efficacy maps: The time in weeks (*T*_*crit*_) at which the tumor volume decreases by 95% from its initial size under treatment with (*γ*_*B*_,*γ*_*A*_). **a** Efficacy map under schedule S1; **b** Efficacy map under schedule S2; **c** Efficacy map under schedule S3. The color columns show the time at which the tumor volume was reduced by 95%

Figure 8 shows “efficacy maps,” namely, the time at which tumor volume was reduced by 95%, under schedules S1, S2 and S3, for a range of the parameters *γ*_*B*_ and *γ*_*A*_. The horizontal axis scales the dose amount of anti-VEGF, *γ*_*B*_, in units of g/cm^3^·day, and the vertical axis scales the dose amount of anti-PD-1, *γ*_*A*_, in unit of g/cm^3^·day. The color columns show the time at which the tumor volume decreased by 95% from its initial size. We see that as *γ*_*A*_ or *γ*_*B*_ increases, the time when the tumor volume was reduced by 95% is decreased. Also, as in the special case of Fig. 6, schedules S2 and S3 reduce significantly the time for the 95% reduction, and the tumor volume reduction by schedule S2 is a little faster than by schedule S3.

We conclude that non-overlapping treatment is far more beneficial than simultaneous treatment, and there is some advantage in injecting anti-PD-1 before anti-VEGF.

**Fig. 9:**
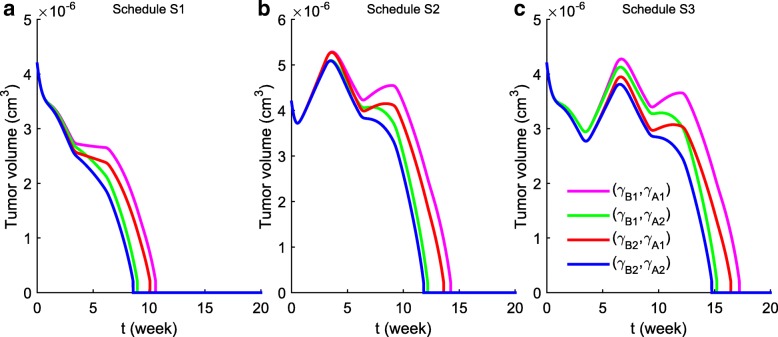
Tumor volume under the schedules S1, S2 and S3 for 4 pairs (*γ*_*B*_,*γ*_*A*_). Here *γ*_*B*1_=9.5×10^−9^g/cm^3^·day,*γ*_*A*1_=1.2×10^−10^g/cm^3^·day,*γ*_*B*2_=9.5×10^−9^g/cm^3^·day,*γ*_*A*2_=1.5×10^−10^g/cm^3^·day. **a** Tumor volume under schedule S1; **b** Tumor volume under schedule S2; **c** Tumor volume under schedule S3

So far we considered the case where anti-VEGF blocks perfusion. We next consider briefly the case where anti-VEGF increases perfusion (as in colon cancer [7, 8]). In this case we have to modify Eqs. (13) and (14) by replacing the term 1/(1+*B*/*K*_*PB*_) by $\left (1+\frac {B}{K_{B}+B}\right)$. In contrast to Figs. 6, 9 shows that simultaneous injection reduces the tumor volume (by 95%) faster than non-overlapping injections: S1 is preferable to S2 and S3, while S2 is preferable to S3. These conclusions hold also when *γ*_*B*_ and *γ*_*A*_ vary in the same range as in Fig. 8.

### Sensitivity analysis

**Fig. 10:**
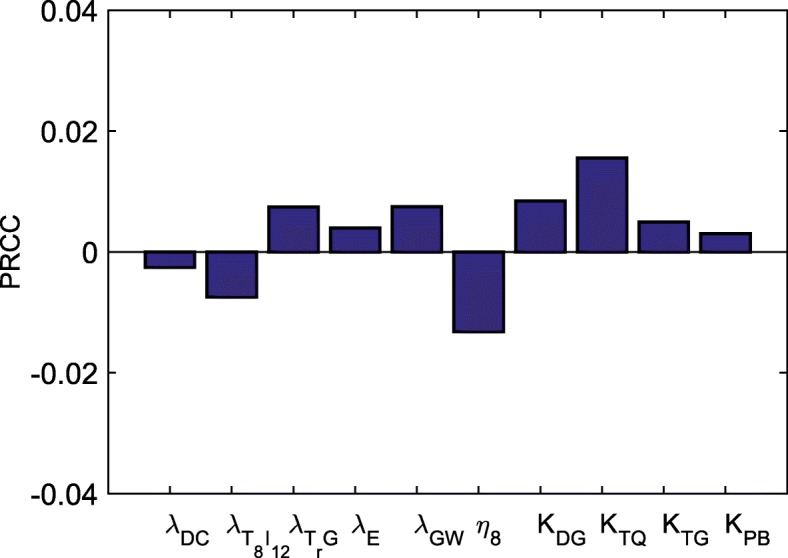
Statistically significant PRCC values (*p*-value <0.01) for tumor volume at day 30

We performed sensitivity analysis with respect to the tumor volume at day 30 for parameters $\phantom {\dot {i}\!}\lambda _{DC}, \lambda _{T_{8}I_{12}}, \lambda _{T_{r}G}, \lambda _{E}, \lambda _{GW}, \eta _{8}, K_{DG}, K_{TQ}, K_{TG}, K_{PB}$. Following the method of [49], we performed Latin hypercube sampling and generated 5000 samples to calculate the partial rank correlation coefficients (PRCC) and the *p*-values with respect to the tumor volume at day 30. In sampling all the parameters, we took the range of each parameter from 1/2 to twice its value in Tables 3 or 4. The results are shown in Fig. 10.

**Table 3:** Summary of parameter values

Notation	Description	Value used	References
*δ*_*D*_	Diffusion coefficient of DCs	8.64×10^−7^cm^2^day^−1^	[59]
*δ*_*T*_	Diffusion coefficient of T cells	8.64×10^−7^cm^2^day^−1^	[59]
*δ*_*E*_	Diffusion coefficient of endothelial cells	8.64×10^−7^cm^2^day^−1^	[59]
*δ*_*C*_	Diffusion coefficient of tumor cells	8.64×10^−7^cm^2^day^−1^	[59]
$\delta _{I_{12}}$	Diffusion coefficient of IL-12	6.05×10^−2^cm^2^day^−1^	[15]
$\delta _{I_{2}}$	Diffusion coefficient of IL-2	9.58×10^−2^cm^2^day^−1^	[15]
$\delta _{T_{\beta }}$	Diffusion coefficient of TGF-β	8.52×10^−2^cm^2^day^−1^	[15]
*δ*_*W*_	Diffusion coefficient of oxygen	0.8 cm^2^day^−1^	Estimated
*δ*_*G*_	Diffusion coefficient of VEGF	8.64×10^−2^cm^2^ day^−1^	[57]
*δ*_*A*_	Diffusion coefficient of anti-PD-L1	4.73×10^−2^cm^2^day^−1^	[15]
*δ*_*B*_	Diffusion coefficient of anit-VGEF	4.70×10^−2^cm^2^day^−1^	Estimated
*σ*_*T*_	Flux rate of *T*_1_ and *T*_8_ cells at the boundary	1 cm^−1^	[59]
*σ*_*E*_	Flux rate of *T*_1_ and *T*_8_ cells at the boundary	1 cm^−1^	[59]
*χ*_*G*_	Chemoattraction coefficient of VEGF	10 cm^5^/g·day	[64, 65]
*λ*_*DC*_	Activation rate of DCs by tumor cells (mice)	17.5day	Estimated
*λ*_*DC*_	Activation rate of DCs by tumor cells (humans)	7.5day	Estimated
$\lambda _{T_{1}I_{12}}$	Activation rate of CD 4^+^ T cells by IL-12	11.65 day^−1^	Estimated
$\lambda _{T_{1}I_{2}}$	Activation rate of CD 4^+^ T cells by IL-2	0.25 day^−1^	[59]
$\lambda _{T_{8}I_{12}}$	Activation rate of CD 8^+^ T cells by IL-12	10.38 day^−1^	Estimated
$\lambda _{T_{8}I_{2}}$	Activation rate of CD 8^+^ T cells by IL-2	0.25 day^−1^	[59]
$\lambda _{T_{r}T_{\beta }}$	Activation rate of Tregs by TGF-β	0.415 day^−1^	Estimated
$\lambda _{T_{r}G}$	Activation rate of Tregs by VEGF	0.083 day^−1^	Estimated
*λ*_*E*_	Growth rate of endothelial cells (mice)	2.77×10^7^cm^3^/g·day	Estimated
*λ*_*E*_	Growth rate of endothelial cells (humans)	2.08×10^7^cm^3^/g·day	Estimated
*λ*_*CW*_	Growth rate of cancer cells (mice)	2.24 day^−1^	Estimated
*λ*_*CW*_	Growth rate of cancer cells (humans)	1.76 day^−1^	Estimated
$\lambda _{I_{12}D}$	Production rate of IL-12 by DCs	2.21×10^−6^day^−1^	[15]
$\lambda _{I_{2}T_{1}}$	Production rate of IL-2 by CD 4^+^ T cells	2.82×10^−8^day^−1^	[15]
$\lambda _{T_{\beta } C}$	Production rate of TGF-β by cancer cells	3.27×10^−10^day^−1^	Estimated
$\lambda _{T_{\beta } T_{r}}$	Production rate of TGF-β by Tregs	5.57×10^−9^day^−1^	[61]
*λ*_*WE*_	Production rate of oxygen by endothelial cells	7×10^−2^/day	[43]
*λ*_*GW*_	Production rate of VEGF by cancer cells	2.21×10^−6^day^−1^	Estimated
*ε*_*T*_	Relative expression of PD-1 in Tregs	0.8	[15]
*ε*_*C*_	Relative expression of PD-L1 in tumor cells	0.01	[15]
*ε*_*G*_	Relative rate of PD-1 promotion by VEGF	1.43×10^6^cm^3^/g	Estimated

**Table 4:** Summary of parameter values

Notation	Description	Value used	References
*d*_*D*_	Death rate of DCs	0.1 day^−1^	[59]
$d_{T_{1}}$	Death rate of CD 4^+^ T cells	0.197day^−1^	[59]
$d_{T_{8}}$	Death rate of CD 8^+^ T cells	0.18day^−1^	[59]
$d_{T_{r}}$	Death rate of Tregs	0.2day^−1^	[61]
*d*_*E*_	Death rate of endothelial cells	0.69 day^−1^	[43]
*d*_*C*_	Death rate of tumor cells	0.17 day^−1^	[59]
$d_{I_{12}}$	Degradation rate of IL-12	1.38 day^−1^	[59]
$d_{I_{2}}$	Degradation rate of IL-2	2.376 day^−1^	[59]
$d_{T_{\beta }}$	Degradation rate of TGF-β	499.066 day^−1^	[15]
*d*_*W*_	Take-up rate of oxygen by cells	1.04 day^−1^	Estimated
*d*_*G*_	Degradation rate of VEGF	12.6 day^−1^	[43]
*d*_*A*_	Degradation rate of anti-PD-L1	0.34 day^−1^	Estimated
*η*_1_	Killing rate of cancer cells by *T*_1_	30.19 cm^3^/g·day	Estimated
*η*_8_	Killing rate of cancer cells by *T*_8_	60.375 cm^3^/g·day	Estimated
*d*_*B*_	Degradation rate of anti-VEGF	0.17 day^−1^	Estimated
*μ*_*PA*_	Blocking rate of PD-1 by anti-PD-1	4.33×10^7^cm^3^/g·day	Estimated
*μ*_*AP*_	Degradation rate of anti-PD-1 in blocking PD-1	4.33×10^7^cm^3^/g·day	Estimated
*μ*_*GB*_	Blocking rate of VEGF by anti-VEGF	2.19×10^7^cm^3^/g·day	Estimated
*μ*_*BG*_	Degradation rate of anti-VEGF in blocking VEGF	1.31×10^8^cm^3^/g·day	Estimated
*K*_*D*_	Half-saturation of CD 4^+^ T cells	4×10^−4^g/cm^3^	[15]
$K_{T_{1}}$	Half-saturation of CD 4^+^ T cells	2×10^−3^g/cm^3^	[15]
$K_{T_{8}}$	Half-saturation of CD 8^+^ T cells	1×10^−3^g/cm^3^	[15]
$K_{T_{r}}$	Half-saturation of Tregs	5×10^−4^g/cm^3^	[15]
*K*_*E*_	Half-saturation of endothelial cells	2.5×10^−3^g/cm^3^	[43]
*K*_*C*_	Half-saturation of tumor cells	0.4 g/cm^3^	[15]
$K_{I_{12}}$	Half-saturation of IL-12	8×10^−10^g/cm^3^	[15]
$K_{I_{2}}$	Half-saturation of IL-2	2.37×10^−11^g/cm^3^	[15]
$K_{T_{\beta }}$	Half-saturation of TGF-β	2.68×10^−13^g/cm^3^	[15]
*K*_*W*_	Half-saturation of oxygen	1.69×10^−4^g/cm^3^	[43]
*K*_*G*_	Half-saturation of VGEF	7×10^−8^g/cm^3^	[43]
$K_{TT_{r}}$	Inhibition of function of T cells by Tregs	5×10^−4^g/cm^3^	[15]
$K^{\prime }_{TQ}$	Inhibition of function of T cells by PD-1-PD-L1	1.68×10^−18^g^2^/cm^6^	Estimated
*K*_*PB*_	Block of anti-PD-1 perfusion by anti-VEGF (mice)	1×10^−8^g/cm^3^	Estimated
*K*_*PB*_	Block of anti-PD-1 perfusion by anti-VEGF (humans)	1×10^−9^g/cm^3^	Estimated
*D*_0_	Density of immature DCs	2×10^−5^ g/ cm^3^	[59]
*T*_10_	Density of naive CD 4^+^ T cells	4×10^−4^ g/ cm^3^	[15]
*T*_80_	Density of naive CD 8^+^ T cells	2×10^−4^ g/ cm^3^	[15]
*E*_*M*_	Carrying capacity of endothelial cells	5×10^−3^ g/ cm^3^	[43]
*C*_*M*_	Carrying capacity of cancer cells	0.8 g/ cm^3^	[59]
*G*_0_	Threshold VEGF concentration	3.65×10^−10^ g/ cm^3^	[43]
$\hat T_{1}$	Density of CD 4^+^ T cells from lymph node	4×10^−3^ g/ cm^3^	[15]
$\hat T_{8}$	Density of CD 8^+^ T cells from lymph node	2×10^−3^ g/ cm^3^	[15]
$\hat E$	Density of endothelial cells from outside of tumor	5×10^−3^ g/ cm^3^	Estimated
*W* ^∗^	Hypoxia threshold oxygen level	1.69×10^−4^ g/ cm^3^	[44]
*W*_0_	Normal threshold oxygen level	4.65×10^−4^ g/ cm^3^	[44]

We see that parameters that promote the killing of cancer cells, such as $\phantom {\dot {i}\!}\lambda _{DC}, \lambda _{T_{8}I_{12}}$ and *η*_8_, are negatively correlated with the tumor volume, while parameters that promote the inhibition of immune cells, such as *K*_*DG*_,*K*_*TQ*_ and *K*_*TG*_, and the proliferation of endothelial cells and Tregs, such as $\phantom {\dot {i}\!}\lambda _{E}, \lambda _{T_{r}G}$ and *λ*_*GW*_, are positively correlated with the tumor volume. We also see that the inhibition of perfusion of the anti-PD-1 drug by anti-VEGF promotes cancer growth, namely, *K*_*PB*_ is positively correlated to the tumor volume. Among the various parameters, *η*_8_ (the killing rate of cancer cells by CD 8^+^ T cells) has the largest impact in reducing tumor volume, and *K*_*TQ*_ (the parameter which increases the inhibition of CD 8^+^ T cells by PD-1-PD-L1) has the largest impact on increasing tumor volume.

## Discussion

Combination therapy for cancer has already shown improved benefits over a single agent therapy [50]. But significant challenges remain, as seen, for example, from the fact that of the successful phase II clinical trials with combination chemotherapy, less then 4% demonstrated, in Phase III, improvement of care within 5 years [11]. It was suggested in [13] that a selection of clinical trials needs to include scientific rationale underlying the data and hypothesis for the combination. Combination clinical trials should be preceded by analysis of the expected interactions between the diverse agents, addressing, for example the following questions:

1. Are the two drugs positively correlated at any amount of dose, and, if so, what should be the most beneficial ratio between the two agents?
2. If the drugs are not always positively correlated, what are the zones of antagonism, i.e., what are the amounts of, and the ratios between, the agents in the combination that may decrease the treatment benefits, and should not be used in clinical trials?
3. If the two drugs are injected intermittently, should they be injected simultaneously or non-overlappingly, and, in the latter case, which drug should be injected first?

In [14] and [15] we addressed the questions (i) and (ii) in the case where one of the drugs is a checkpoint inhibitor (anti-PD-1), and the second drug is a cancer vaccine [14] or a BRAF-inhibitor [15]. In the present paper we addressed the question (iii) when the two drugs are anti-PD-1 and anti-VEGF. Anti-VEGF is known to block the perfusion of chemotherapy in melanoma [3], breast cancer [4, 5, 9] and ovarian cancer [6], but to increase perfusion in colon cancer [7].

We developed a mathematical model and simulations for *in silico* clinical trials that addressed the complexity of interactions among the two drugs. The model is represented by a system of PDEs that includes the most relevant cells and cytokines associated with the treatment. The simulations show that in the case where anti-VEGF decreases the perfusion of the anti-PD-1, the time it takes to reduce tumor volume by 95% is much shorter when the injections of the two drugs are non-overlapping than when the injections are given at the same time. Furthermore, in the non-overlapping treatment, if we inject the anti-PD-1 first we get the 95% -reduction somewhat faster than if we inject anti-VEGF first.

On the other hand, in the case when anti-VEGF increases the perfusion of anti-PD-1, a treatment with simultaneous infections is more beneficial than a treatment with non-overlapping injections.

The optimal scheduling in combination therapy for cancer was considered in several publications, both in terms of toxicity [51] and efficacy [52–54]. The present paper considered the efficacy question by a mathematical model. The method and results of the paper can be extended to other combinations, for example to a chemotherapeutic agent combined with anti-VEGF, and they could play an important role in the design of clinical trials, where scheduling strategies may significantly affect the outcome.

## Conclusions

The design of cancer clinical trials with combination of two drugs should take into account the potential interactions between the two agents, which may suggest an optimal scheduling strategy in the administration of the drugs. Mathematical models can play a useful role in this process. This was illustrated in the case where the drugs are anti-VEGF and anti-PD-1. Anti-VEGF increases the activation of anti-cancer cells, but it also modifies the perfusion of other drugs. Using a mathematical model we showed that a non-overlappling adminstration of the two drugs is more effective in reducing tumor volume than simultaneous administration of the drugs in the case where anti-VEGF degrades perfusion, as it occurs in some cancers, while the opposite is true when perfusion is enhanced by anti-VEGF. The mathematical methodology developed in this paper could be extended to treatment with other combinations of drugs.

## Appendix

### Parameter estimation

#### Half-saturation

In an expression of the form $Y\frac {X}{K_{X}+X}$ where *Y* is activated by *X*, the parameter *K*_*X*_ is called the half-saturation of *X*.

We assume that if the average density (or concentration) of *X*,

$$\frac{\int X dx}{\int dX}, $$

converges to a steady state *X*_0_, then

$$\frac{X_{0}}{K_{X}+X_{0}} $$

is not “too small” and not “too close” to 1, and for definiteness we take

$$\frac{X_{0}}{K_{X}+X_{0}}=\frac{1}{2} $$

so that

28 $$  K_{X}=X_{0}.  $$

To estimate parameters, we assume that all the average densities and concentrations converge to their respective steady states, and thus use Eq. (28) in each of the steady state equations of the model.

#### Diffusion coefficients

We use the following relation for estimating the diffusion coefficient of a protein *p* [55]:

$$\delta_{p}=\frac{M^{1/3}_{G}}{M^{1/3}_{p}}\delta_{G}, $$

where *M*_*G*_ and *δ*_*G*_ are respectively the molecular weight and diffusion coefficient of VEGF, *M*_*p*_ is the molecular weight of *p*, and *M*_*G*_=24kDa [56] and *δ*_*G*_=8.64×10^−2^cm^2^ day^−1^ [57]. Since, *M*_*B*_=149kDa (bevacizumab), we get *δ*_*B*_=4.70×10^−2^cm^2^ day^−1^. The diffusion coefficient of oxygen in the extracellular matrix (ECM) in the range of 7×10^−6^−2×10^−5^cm^2^/s [58]; we take it to be *δ*_*W*_=0.8cm^2^/day.

#### Eq. (2).

From the steady state of Eq. (2) (more precisely, by setting to zero the RHS of Eq. (2)), we get $\lambda _{DC}D_{0}\frac {C}{K_{C}+C}\cdot \frac {1}{1+G/K_{DG}}=d_{D}D$, where by [15], *d*_*D*_=0.1/day, *C*=*K*_*C*_=0.4g/cm^3^,*D*=*K*_*D*_=4×10^−4^g/cm^3^,*D*_0_=2×10^−5^g/cm^3^. We assume that *K*_*DG*_=4*K*_*G*_ where *K*_*G*_=7×10^−8^g/cm^3^ [43]; hence *λ*_*DC*_=2.5*d*_*D*_*D*/*D*_0_=5/day. For simplicity we had assumed that the source of inactive dendritic cells, *D*_0_, is constant. However, this source actually decreases as more dendritic cells become activated. We take this into count by increasing *λ*_*DC*_, taking *λ*_*DC*_=17.5/day in mice and *λ*_*DC*_=7.5/day in humans.

#### Eqs. (3) and (4).

We assume that in steady state, *Q*/*K*_*TQ*_=2 (the value of *K*_*TQ*_ is derived in the estimates for Eqs. (13)-(17)). We also assume that *K*_*TG*_=4*K*_*G*_ where *K*_*G*_=7×10^−8^g/cm^3^ [43]. From the steady state of Eq. (3), we get

$$\left(\lambda_{T_{1}I_{12}}T_{10}\cdot\frac{1}{2}\cdot\frac{1}{2}\cdot\frac{4}{5}+\lambda_{T_{1}I_{2}}T_{1}\cdot\frac{1}{2}\right)\cdot\frac{1}{3}-d_{T_{1}}T_{1}=0, $$

where, by [15], $\lambda _{T_{1}I_{2}}=0.25$/day, $d_{T_{1}}=0.197$/day, *T*_10_=4×10^−4^g/cm^3^ and $T_{1}=K_{T_{1}}=2\times 10^{-3} \mathrm {g}/\text {cm}^{3}$. Hence $\lambda _{T_{1}I_{12}}=11.65/\text {day}$.

From the steady state of Eq. (4), we have

$$\left(\lambda_{T_{8}I_{12}}T_{80}\cdot\frac{1}{2}\cdot\frac{1}{2}\cdot\frac{4}{5}+\lambda_{T_{1}I_{2}}T_{8}\cdot\frac{1}{2}\right)\cdot\frac{1}{3}-d_{T_{8}}T_{8}=0 $$

where by [15], $\lambda _{T_{8}I_{2}}=0.25$/day, $d_{T_{8}}=0.18$/day, $T_{80}=2\times 10^{-4} \mathrm {g}/\text {cm}^{3}, T_{8}=K_{T_{8}}=1\times 10^{-3} \mathrm {g}/\text {cm}^{3}$. Hence $\lambda _{T_{8}I_{12}}=10.38/\text {day}$.

As in the case of Eq. (2), we had ignored the decrease in the sources *T*_10_ and *T*_80_ as more of these cells become activated, but we also ignored the contribution of the flux of T cells at the tumor’s boundary. We assume that the flux of T cells compensates for decrease in the source of the inactive T cells, and retain the above values of $\lambda _{T_{1}I_{12}}$ and $\lambda _{T_{8}I_{12}}$. The same considerations apply in the case of *T*_*r*_.

#### Eq. (5).

We assume that TGF-β activates Tregs more than VEGF does, and take $\lambda _{T_{r}T_{\beta }}=5\lambda _{T_{r}G}$. From the steady state of Eq. (5), we get, $(\lambda _{T_{r}T_{\beta }}\cdot \frac {1}{2}+\lambda _{T_{r}G}\cdot \frac {1}{2})T_{10}-d_{T_{r}}T_{r}=0$, where $T_{10}=1\times 10^{-3} \mathrm {g}/\text {cm}^{3}, T_{r}=K_{T_{r}}=5\times 10^{-4} \mathrm {g}/\text {cm}^{3}$ [59], and $d_{T_{r}}=0.2$/day [59]. Hence $\lambda _{T_{r}G}=0.083/\text {day}$ and $\lambda _{T_{r}T_{\beta }}=0.415$/day.

#### Eq. (6).

By [43], *d*_*E*_=0.69/day, *E*_*M*_=5×10^−3^g/cm^3^,*K*_*E*_=2.5×10^−3^g/cm^3^,*G*_0_=3.65×10^−10^. We take *E*_*M*_=2*K*_*E*_=5×10^−3^g/cm^3^. From the steady state of Eq. (6), we get *λ*_*E*_=2*d*_*E*_/(*K*_*G*_−*G*_0_)=1.98×10^7^cm^3^/g·day. Assuming that the threshold *G*_0_ (which was taken to be constant) is actually increasing with the progression of the cancer in the control case (no drugs), we increase *λ*_*E*_, taking *λ*_*E*_=2.77×10^7^cm^3^/g·day in mice and *λ*_*E*_=2.08×10^7^cm^3^/g·day in humans.

#### Eq. (7).

We take *d*_*C*_=0.17day^−1^,*C*_*M*_=0.8g/cm^3^ [59] and *λ*_*CW*_=1.6/day [60]. In the steady state of the control case (no drugs), we assume that *C* is approximately 0.4 g/cm^3^, and *W*=*W*_0_=*K*_*W*_=1.69×10^−4^g/cm^3^ (see the estimates for Eq. (11)). From the steady state of Eq. (7) in the control case we have,

$$\frac{1}{2}\lambda_{CW}K_{W}/W_{0}-\eta_{1}K_{T_{1}}-\eta_{8}K_{T_{8}}-d_{C}=0, $$

where $K_{T_{1}}=2\times 10^{-3} \mathrm {g}/\text {cm}^{3},K_{T_{8}}=1\times 10^{-3} \mathrm {g}/\text {cm}^{3}$; we take *η*_8_=2*η*_1_; hence $\eta _{8}=(\lambda _{C}K_{W}/(2W_{0})-d_{C})/(2K_{T_{8}})=60.375 \text {cm}^{3}/\mathrm {g}\cdot \text {day}$ and *η*_1_=30.19cm^3^/g·day. Since in the control case the tumor grows, we increase the growth rate of cancer cells taking *λ*_*CW*_=2.24/day in mice and *λ*_*CW*_=1.76/day in humans.

#### Eq. (10).

From the steady state of Eq. (10) we have, $\lambda _{T_{\beta } C}C+\lambda _{T_{\beta } T_{r}}T_{r}=d_{T_{\beta }}T_{\beta }$, where $T_{r}=K_{T_{r}}=5\times 10^{-4} \mathrm {g}/\text {cm}^{3},C=K_{C}=0.4 \mathrm {g}/\text {cm}^{3}$, and, by [15], $T_{\beta }=K_{T_{\beta }}=2.68\times 10^{-13} \mathrm {g}/\text {cm}^{3}$ and $d_{T_{\beta }}=499.07 \text {day}^{-1}$, and by [61], $\lambda _{T_{\beta } T_{r}}=5.57\times 10^{-9}$/day. Hence $\lambda _{T_{\beta } C}=3.27\times 10^{-10}$/day.

#### Eq. (11).

From steady state of Eq. (11) we get, *λ*_*WE*_*E*−*d*_*W*_*W*=0, where *λ*_*WE*_=7×10^−2^/day [43], *W*=*K*_*W*_=1.69×10^−4^g/cm^3^ [43], *E*=*K*_*E*_=2.5×10^−3^g/cm^3^. Hence, *d*_*W*_=*λ*_*WE*_*E*/*W*=1.04/day.

#### Eq. (12).

From steady state of Eq. (12) we get, *λ*_*GW*_*C*−*d*_*G*_*G*=0, where *d*_*G*_=12.6/day [43], *G*=*K*_*G*_=7×10^−8^g/cm^3^ [43], *C*=*K*_*C*_=0.4g/cm^3^. Hence, *λ*_*GW*_=2.21×10^−6^/day.

#### Eqs. (13)-(17).

By [15], *ρ*_*P*_=2.49×10^−7^,*ρ*_*L*_=5.22×10^−7^,*ε*_*T*_=0.8,*ε*_*C*_=0.01. We assume that *ε*_*G*_=0.1/*K*_*G*_=1.43×10^6^cm^3^/g. From Eqs. (13)-(16) we get,

$$\begin{array}{@{}rcl@{}} K_{P_{1}}=P_{1}&=&\rho_{P}(T_{1}+\varepsilon_{T} T_{r})\\ &=&(2.49\!\times\! 10^{-7})\times\left[2\!\times\! 10^{-3} \,+\,0.8\!\times\! (5 \times 10^{-4})\right]\\ &=&5.98\times 10^{-10}\mathrm{g}/\text{cm}^{3}, \end{array} $$

$$\begin{array}{@{}rcl@{}} K_{P_{8}}=P_{8}&=&\rho_{P}T_{8}(1+\varepsilon_{G} G)\\ &=&\left(2.49\times 10^{-7}\right)\times\left(1\times 10^{-3}\right)\times (1+0.1)\\ &=&2.74\times 10^{-10}\mathrm{g}/\text{cm}^{3}, \end{array} $$

and

$$\begin{array}{@{}rcl@{}} K_{L}=L&=&\rho_{L}(T_{1}+T_{8}+\varepsilon_{T} T_{r}+\varepsilon_{C} C)\\ &=&\left(5.22\times 10^{-7}\right)\times\left[3.4\times 10^{-3} +0.01\times 0.4\right]\\ &=&3.86\times 10^{-9}\mathrm{g}/\text{cm}^{3}. \end{array} $$

In steady state with $\phantom {\dot {i}\!}P=K_{P}=K_{P_{1}}+K_{P_{8}},L=K_{L},Q=K_{Q}$ and *G*=*K*_*G*_ we have, by Eq. (17), *K*_*Q*_=*σK*_*P*_*K*_*L*_. We take $K_{TQ}=K_{Q}=\frac {1}{2}\sigma K_{P}K_{L}$. Hence, $Q/K_{TQ}=PL/\left (\frac {1}{2}K_{P}K_{L}\right)$ and

$$\frac{1}{1+Q/K_{TQ}}=\frac{1}{1+PL/\left(\frac{1}{2}K_{P}K_{L}\right)}=\frac{1}{1+PL/K'_{TQ}}, $$

where $K^{\prime }_{TQ}=\frac {1}{2}K_{P}K_{L}=\frac {1}{2}\times \left (5.98\times 10^{-10}+2.74\times 10^{-10}\right)\times \left (3.86\times 10^{-9}\right)=1.68\times 10^{-18} \mathrm {g}^{2}/\text {cm}^{6}$.

#### Eqs. (18)-(19).

We take *d*_*A*_=0.34day^−1^ and assume that 10% of *A* is used in blocking PD-1, while the remaining 90% degrades naturally. Hence,

$${{} \begin{aligned} \mu_{AP}\,=\,\frac{d_{A}}{9P}\,=\,\frac{0.34}{9\times (8.715\!\times\! 10^{-10})}\,=\,4.33\!\times\! 10^{7}\: \text{cm}^{3}/\mathrm{g}\cdot \text{day}. \end{aligned}} $$

Since the molecular mass of anti-PD-1 (32kDa [62]) approximates the molecular mass of PD-1 (20.5 - 40 kDa [62]), we take *μ*_*PA*_=*μ*_*AP*_=4.33×10^7^ cm^3^/g·day.

By [63], the half-life of anti-VEGF is 2.82-4.58 days; we take it to be 4 days, so that $d_{B}=\frac {\text {ln} 2}{4}=0.17 \text {day}^{-1}$. We assume that 90% of *B* is depleted in blocking of VEGF, while the remaining 10% degrades naturally. Hence,

$$\mu_{BG}=\frac{9d_{B}}{G}=\frac{9\time 0.17}{7\times 10^{-8}}=2.19\times 10^{7}\: \text{cm}^{3}/\mathrm{g}\cdot \text{day}. $$

The molecular mass of anti-VEGF is approximately 6 times larger than that of VEGF, so we take *μ*_*GB*_=6*μ*_*BG*_=1.31×10^8^ cm^3^/g·day.

### Computational method

We employ a moving mesh method [48] to numerically solve the free boundary problem for the tumor proliferation model. To illustrate this method, we take Eq. (7) as an example and rewrite it in the following form:

29 $$ \begin{aligned} \frac{\partial C(r,t)}{\partial t}=\delta_{C}\Delta C(r,t)-div(\mathbf{u}C)+ F, \quad  \end{aligned}  $$

where *F* represents the term in the right hand side of Eq. (7). Let $r_{i}^{k}$ and $C_{i}^{k}$ denote numerical approximations of i-th grid point and $C(r_{i}^{k},n\tau)$, respectively, where *τ* is the size of the time-step. The discretization of Eq. (29) is derived by the fully implicit finite difference scheme:

30 $$ \begin{aligned} \frac{C_{i}^{k+1}-C_{i}^{k}}{\tau}=&\delta_{C}\left(C_{rr}+\frac{2}{r_{i}^{k}}C_{r}\right)-\left(\frac{2}{r_{i}^{k+1}}u_{i}^{k+1}+u_{r}\right)C_{i}^{k+1}\\&-u_{i}^{k+1}C_{r}+F_{i}^{k+1}, \end{aligned}  $$

where $C_{r}=\frac {h_{-1}^{2}C_{i+1}^{k+1}-h_{1}^{2}C_{i-1}^{k+1}-(h_{1}^{2}-h_{-1}^{2})C_{i}^{k+1}}{h_{1}(h_{-1}^{2}-h_{1}h_{-1})},C_{rr}=2\frac {h_{-1}C_{i+1}^{k+1}-h_{1}C_{i-1}^{k+1}+(h_{1}-h_{-1})C_{i}^{k+1}}{h_{1}(h_{1}h_{-1}-h_{-1}^{2})}$,$u_{r}=\frac {h_{-1}^{2}u_{i+1}^{k+1}-h_{1}^{2}u_{i-1}^{k+1}-(h_{1}^{2}-h_{-1}^{2})u_{i}^{k+1}}{h_{1}(h_{-1}^{2}-h_{1}h_{-1})},h_{-1}=r_{i-1}^{k+1}-r_{i}^{k+1}$ and $h_{1}=r_{i+1}^{k+1}-r_{i}^{k+1}$. The mesh moves by $r_{i}^{k+1}=r_{i}^{k}+u_{i}^{k+1}\tau $, where $u_{i}^{k+1}$ is solved by the velocity equation. To deal with the different scales of the variables in the simulations, we first non-dimensionalized the model and then applied the above method.
